# Copper Deficiency, Lead, and Paraoxonase

**DOI:** 10.1289/ehp.10151

**Published:** 2007-07

**Authors:** Leslie M. Klevay

**Affiliations:** University of North Dakota, School of Medicine and Health Sciences, Grand Forks, North Dakota, E-mail: leslie_klevay@und.nodak.edu

Li et al. (2006) measured paraoxonase 1 (PON1) in workers and found an inverse association between lead exposure and enzyme activity. This observation compliments some epidemiology and related experiments with animals, because low paraoxonase activity is associated with diabetes mellitus, familial hypercholesterolemia, ischemic heart disease, and metabolic syndrome (Klevay 2004). Paraoxonase, although studied most extensively because of its ability to detoxify organophosphate insecticides (James 2006; van Himbergen et al. 2006), has drawn increasing attention because it hydrolyzes homocysteine thiolactone, a vascular toxin that inhibits copper enzymes (Klevay 2006).

Lead intoxication has many manifestations (Fischbein 1998), lesser-known of which is induction of copper deficiency (Klauder and Petering 1977). Rats deficient in copper have an approximately 28% decrease in paraoxonase activity (Klevay 2004). These observations are consonant with the decrease in superoxide dismutase (SOD) associated with occupational exposure to lead (Ito et al. 1985) because this enzyme also depends on adequate copper nutriture for activity (Linder and Goode 1980; Owen 1981). Thus, SOD is an index of copper nutriture in humans (Uauy et al. 1985).

Li et al. (2006) stated that “the mechanism by which heavy metals inhibit serum PON1 activity is still not clear.” It seems likely that lead interferes with copper utilization in the workers, leading to low copper nutritional status (Li et al. 2006). Low copper status has been related to a large variety of adverse cardiovascular phenomena in both animals and people; in this context, the most important are hypercholesterolemia, hypertension, and impaired oxidative defense (Klevay 2000, 2002).

Are there unpublished copper data on the workers, or can they be reexamined to test this copper hypothesis? Plasma copper and ceruloplasmin are not likely to be useful because they are increased by inflammation (Pepys 1996) and may be falsely high. Extracellular SOD may be helpful because it is sensitive to low copper status (Johnson et al. 2005), and low values have been associated with atherosclerosis in humans (Landmesser et al. 2000; Wang et al. 1998).
